# Short-Term Eating Preference of Beef Cattle Fed High Forage or High Grain Diets Supplemented with 3-Nitrooxypropanol

**DOI:** 10.3390/ani10010064

**Published:** 2019-12-30

**Authors:** Chanhee Lee, Seon-Ho Kim, Karen Beauchemin, Pietro Celi, Stéphane Duval

**Affiliations:** 1 Department of Animal Sciences, Ohio Agricultural Research and Development Center, The Ohio State University, Wooster, OH 44691, USA; mhs0425@hanmail.net; 2 Lethbridge Research and Development Centre, Agriculture and Agri-Food Canada, Lethbridge, AB T1J4B1, Canada; karen.beauchemin@canada.ca; 3 DSM Nutritional Products, 4303 Kaiseraugst, Switzerland; Pietro.celi@dsm.com; 4 DSM Nutritional Products, 68305 Saint Louis, France; Stephane.duval@dsm.com

**Keywords:** 3-nitrooxypropanol, eating preference, beef steers

## Abstract

**Simple Summary:**

3-Nitrooxypropanol is an effective methane-mitigating feed additive. However, a decrease in feed intake is frequently observed in beef cattle fed a diet supplemented with 3-nitrooxypropanol. Thus, a study was conducted to determine the short-term preference of beef cattle offered diets supplemented with 3-nitrooxypropanol. When given a choice, the cattle initially showed less preference for a diet supplemented with 3-nitrooxypropanol, suggesting potential changes in organoleptic properties of the diet. However, the animals acclimatized to the supplemented diet rapidly such that there was no preference between a diet with and without 3-nitrooxypropanol within a week. Therefore, the change in the organoleptic properties of a diet supplemented with 3-nitrooxypropanol does not appear to be the main reason for reduced feed intake of beef cattle.

**Abstract:**

Two experiments were conducted to examine eating preference of beef cattle for diets with or without the investigative enteric methane inhibitor 3-nitrooxypropanol (3-NOP). Nine beef steers were housed in individual stalls, each equipped with two feed bunks. The first experiment (Exp. 1) was conducted with a high forage diet and each animal received a diet without 3-NOP (CON) in one bunk and a diet with 3-NOP (dNOP) in the other bunk. The second study (Exp. 2) was conducted with the same animals about 6 months after Exp. 1 where a high grain diet without (CON) or with 3-NOP (dNOP) was offered. In Exp. 1, animals initially preferred CON compared with dNOP. Feed consumption from 0 to 3, 3 to 6, and 6 to 12 h after feeding was lower for dNOP compared with CON. However, dry matter intake (DMI) and feed consumption of dNOP gradually increased during Exp. 1 such that there was no preference between CON and dNOP on day 7. In Exp. 2, there was no preference for or against dNOP. Average DMI was greater for dNOP vs. CON, but interactions between diet and day for DMI and feed consumption rates indicated that daily preference between CON and dNOP was variable. In conclusion, beef steers initially detected a difference between CON and dNOP and selected in favor of CON rather than dNOP when they had not previously been exposed to 3-NOP. However, the animals rapidly acclimatized to a diet with 3-NOP (Exp. 1) and showed no eating preference between CON and dNOP within 7 days. This lack of preference was maintained throughout Exp. 2 when the same animals were fed a high grain diet.

## 1. Introduction

3-Nitrooxypropanol (3-NOP) is an investigational feed additive for ruminants that has been shown to reduce enteric methane production. Studies have shown that: (1) 3-NOP significantly decreased methane production regardless of type of diet fed (i.e., high forage and high grain diets, according to Vyas et al. [1]); and (2) the effectiveness of 3-NOP for enteric methane mitigation was consistent among studies and persistent [1,2]. However, a decrease in feed intake is often observed in beef cattle fed a diet with 3-NOP without negative effects on production performance (growth and carcass characteristics [1,3]). The decrease in feed intake could be caused by improved energy status when methane production is decreased (e.g., increased rumen propionate production [4,5]). Another factor that affects dry matter intake (DMI) is a change in organoleptic property of the diet when a feed additive is included [6]. Decreased feed intake when feeding supplemental 3-NOP has often been explained by metabolic effects because 3-NOP typically increases rumen propionate concentration when lowering enteric methane production [1,7]. However, changes in the organoleptic property of a diet due to 3-NOP supplementation cannot be ignored and this aspect has not yet been studied. Based on the observed linear decrease in DMI with increasing level of 3-NOP in a high forage diet reported by Romero-Perez et al. [3], a potential palatability issue due to 3-NOP supplementation of diets can be hypothesized. Furthermore, if inclusion of 3-NOP alters the organoleptic property of a diet, thereby affecting DMI of cattle, the degree of change in DMI could be different depending upon type of diet (e.g., backgrounding and finishing diets). For example, when nitrate feed additive was fed to beef cattle during a backgrounding phase followed by finishing phase (i.e., continuous feeding of nitrate with a high forage diet for 70 d followed by a high grain diet for about 160 d to beef cattle), supplemental nitrate altered DMI and particle size of orts, indicating that inclusion of nitrate altered organoleptic properties of the diets [6,8]. However, in that study the degree of change in DMI and particle size of orts was different between the high forage and high grain diet, suggesting that the type of diet and adaptation can affect the degree of change in organoleptic properties of diets when feed additives are used.

Therefore, the objective of the current study was to determine eating preference of beef cattle fed a high forage diet supplemented with and without 3-NOP, followed by a high grain diet with and without 3-NOP. We hypothesized that if 3-NOP negatively alters the organoleptic properties of a diet, then a diet containing 3-NOP would be less preferable compared with a diet without 3-NOP when animals are given a choice. Additionally, the animals would acclimatize to the diet containing 3-NOP over time, such that no preference would be observed when 3-NOP was provided to cattle fed a high grain diet after receiving a high forage diet supplemented with 3-NOP.

## 2. Materials and Methods 

### 2.1. Animals and Experimental Procedure

A total of 9 Angus beef steers (average ± SD; BW, 349 ± 9.0 kg at the beginning of the experiment) were used in this experiment and housed in individual stalls (3 m × 4.9 m). The eating preference was determined using a split-plate choice evaluation method [9]. Each stall had two feed bunks that were identical in size, and a source of water. The first experiment (Exp. 1) was conducted with a high forage diet as a basal diet (CON; Table 1). The treatment diet was the basal diet supplemented with 3-NOP (dNOP). The 3-NOP product (silicon dioxide and 3-NOP) was included to provide 3-NOP at 100 mg/kg dietary dry matter (DM). The 3-NOP product was initially blended with a concentrate mix and stored at the barn. Another concentrate mix without 3-NOP was prepared and stored for CON. The concentrate was mixed with forage for the total mixed ration (TMR) and offered every morning (09:00) once daily.

The basal diet was formulated to meet the nutrient requirements of growing beef cattle according to National Academies of Sciences, Engineering, and Medicine (NASEM) [10]. The experiment was conducted for 21 d, with the first 14 d as diet adaptation during which the CON diet was delivered to both feed bunks for each individual animal. Dry matter intake from each bunk per animal for the last 7 d of the diet adaptation was monitored as a pre-preference period. The pre-preference period was followed by a 7 d preference period where the CON diet was delivered to one feed bunk and the dNOP diet was delivered to the other bunk in each individual stall to evaluate eating preference of animals. The location of the diets (CON and dNOP), i.e., left and right bunk in each stall, was switched daily to remove potential bunk preference of animals. The same amount of feed was allocated to both bunks and each was provided for ad libitum intake. In the first 7 d of the diet adaptation, feed intake of individual animals was monitored to determine ad libitum intake (about 5% refusal) for the pre-preference and preference periods. To avoid a potential lack of feed in a feed bunk during the preference period (i.e., if preference for a particular diet was high), adequate feed was allocated to each feed bunk (1.8 × ad libitum feed intake ÷ 2 per bunk).

Diet samples were collected on 2 non-consecutive days weekly and composited by period (pre-preference and preference) and refusal samples in each bunk were collected and composited by week, bunk, and animal. Diet and refusal samples were dried (55 °C for 72 h) to calculate DMI from each bunk and total DMI for individual animals. Composited diet samples were ground to pass a 1 mm screen (Wiley Mill; Arthur A. Thomas Co., Philadelphia, PA) and the ground samples were submitted to Rock River Laboratory (Watertown, WI) for standard chemical analyses shown in Table 1 (https://www.rockriverlab.com/pages/Animal-Nutrition.php).

Feed consumption rate of individual animals for each bunk was determined during the preference period. Daily feed consumption rate (as-fed basis) by time after feeding was determined for 7 d for the following intervals after feeding: 0 to 3 h, 3 to 6 h, 6 to 12 h, and 12 to 24 h. This was done by weighing the feed remaining in each bunk at 3, 6, 12, and 24 h after feeding.

After finishing Exp. 1, the second experiment (Exp. 2) used the same animals (average ± SD; 598 ± 42 kg at the beginning of the experiment) and was conducted using the same experimental design and procedures as in Exp. 1 (i.e., a split-plate choice evaluation method) except that a high grain diet (CON; Table 1) and a high grain diet supplemented with 3-NOP (100 mg/kg of dietary DM; dNOP) were fed to the animals. In addition, the preference period lasted for 8 d. The diet was formulated to meet the nutrient requirements of finishing beef cattle according to NASEM [10].

After the end of Exp. 1 and before starting Exp. 2, the animals were used in another experiment for 4 months [11]. That study was a repeated 3 × 3 Latin square design where the animals received a high forage diet (control), a high forage diet supplemented with 3-NOP (100 mg/kg dietary DM), or the control diet with 3-NOP being infused into the rumen. After the experiment, the animals were not involved in any experiments for about 2 months until the start of the current Exp. 2. During the 2 months, all animals received a transitioning diet without 3-NOP gradually switching them from the high forage diet to the high grain diet.

### 2.2. Calculations and Statistical Analyses

Dry matter intake of individual animals from each bunk was measured daily and daily total DMI of individual animals was obtained by summing the DMI from both bunks per animal throughout the experiment. Feed consumption rates were calculated on an as-fed basis by subtracting the amount of feed offered by the amount of feed remaining in each bunk at 3, 6, 12, and 24 h after feeding.

Data were analyzed using the MIXED procedure of SAS (version 9.3; SAS Institute Inc., Cary, NC, USA). Data of DMI during the pre-preference period (7 d) and preference period (7 d for Exp. 1 and 8 d for Exp. 2) were compared using a model including animal-within period and error term (random effect) and period, day, and period × day (fixed effects). Dry matter intakes from the two feed bunks per animal during the pre-preference period were compared with a model that included animal and error term as random effect and bunk, day (repeated), and bunk × day as fixed effect. The same model was used to compare DMI of diets during the preference period (CON vs. dNOP) except that bunk was replaced with diet. Feed consumption rates for CON and dNOP at 0 to 3, 3 to 6, 6 to 12, and 12 to 24 h after feeding were compared with the model described above. During the preference period, changes in proportion of dNOP consumed in total intake over 7 d were analyzed (linear regression) using the REG procedure of SAS. In addition, differences in proportion between CON and dNOP for individual days during the preference period were analyzed. For this comparison, proportion of CON consumption was subtracted from the proportion of dNOP consumption and the difference was compared to 0 using the same model (MIXED procedure) used for feed consumption data. All data are presented as least square means. Statistical differences were declared at *p* < 0.05. Differences between treatments with 0.05 < *p* < 0.10 were considered a tendency toward significance.

## 3. Results

Dry matter intake of animals between the pre-preference and preference period was not different (*p* ≤ 0.71) in Exp. 1 and 2 (Table 2). A significant interaction of period by day in Exp. 1 was observed (*p* < 0.01) and further investigation of the interaction indicated that DMI gradually increased during the pre-preference period while DMI during the preference period was relatively constant. In Exp. 2, an interaction of period by day (*p* < 0.01) was also observed. This interaction occurred because DMI of animals on day 2 dropped and then gradually returned to the normal intake level during the pre-preference period and DMI during the preference period was relatively constant (data not shown). The decrease in DMI on day 2 in the pre-preference period occurred for all animals for unidentified reasons and might have been caused by environmental changes.

**Figure 1:**
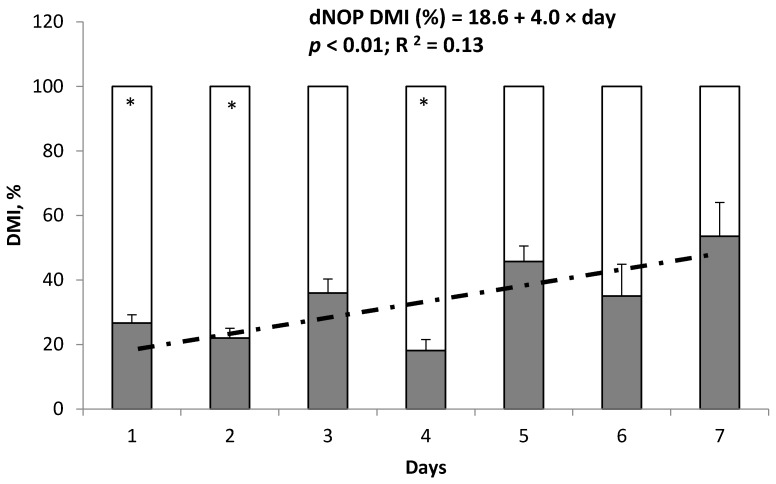
Proportions of DMI of beef steers (*n* = 9) fed a high forage diet without (white portion of the bars) or with 3-NOP (black portion of the bars) during the preference period (Exp. 1). The symbol * indicates that the proportion of the CON diet consumed minus proportion of the 3-NOP diet consumed within day is not equal to 0 (*p* < 0.05). Day 1 to 7 corresponds to the 7 d preference period. The dashed line indicates the linear regression of intake of 3-NOP diet, as a percentage of total daily dry matter intake (dNOP DMI, %), as a function of day during the preference period.

**Figure 2:**
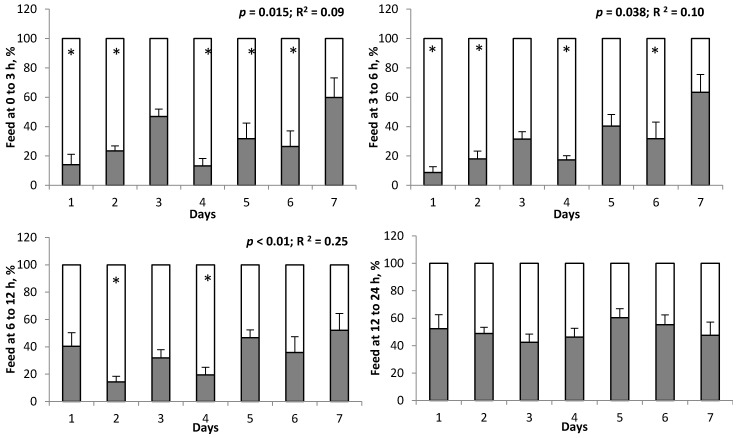
Proportions of a high forage diet without (white portion of the bars) or with 3-NOP (black portion of the bars) consumed by beef steers at 0 to 3, 3 to 6, 6 to 12, and 12 to 24 h during the preference period (*n* = 9; Exp. 1). Day 1 to 7 corresponds to the 7 d preference period. The symbol * indicates that the proportion of CON diet consumed minus proportion of 3-NOP diet consumed within day is not equal to 0 (*p* < 0.05). If *P* and R^2^ values are shown, a significant linear change in intake of 3-NOP diet, as a percentage of total dry matter intake, occurred over the 7 day preference period.

During the pre-preference period, DMI between the 2 feed bunks in Exp. 1 and 2 was not different (*p* ≥ 0.17; Table 3). In Exp. 1, when both CON and dNOP were delivered to individual animals during the preference period, DMI was lower (*p* < 0.01) for dNOP compared with CON. The proportion of dNOP consumed vs. CON was lower (*p* < 0.01) from 0 to 12 h after feeding (Table 4). At 12 to 24 h after feeding, no difference between CON and dNOP (*p* = 0.69) was observed. Significant interactions of diet by day for DMI and feed consumption (*p* < 0.01) were observed during the preference period because the difference in DMI between CON and dNOP diminished gradually (*p* < 0.01; R^2^ = 0.13; Figure 1) over the 7-d preference period. A similar pattern of feed consumption during the preference period was observed from 0 to 3, 3 to 6, 6 to 12, and 12 to 24 h after feeding where the proportion of dNOP consumption for the first 12 h after feeding was gradually increased (*p* ≤ 0.038) over 7 d (Figure 2).

**Figure 3:**
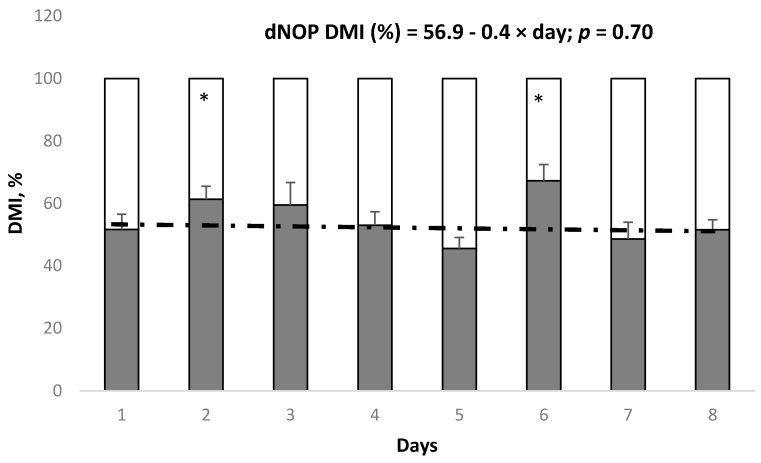
Proportions of DMI of animals (*n* = 9) fed a high grain diet without (white portion in the bars) or with 3-NOP (black portions in the bars) during the preference period (Exp. 2). The symbol * indicates that the proportion of CON diet consumed minus proportion of 3-NOP diet consumed within day is not equal to 0 (*p* < 0.05). Day 1 to 8 corresponds to the 8 d preference period. The dashed line indicates the linear regression of intake of 3-NOP diet, as a percentage of total daily dry matter intake (dNOP DMI, %), as a function of day.

**Figure 4:**
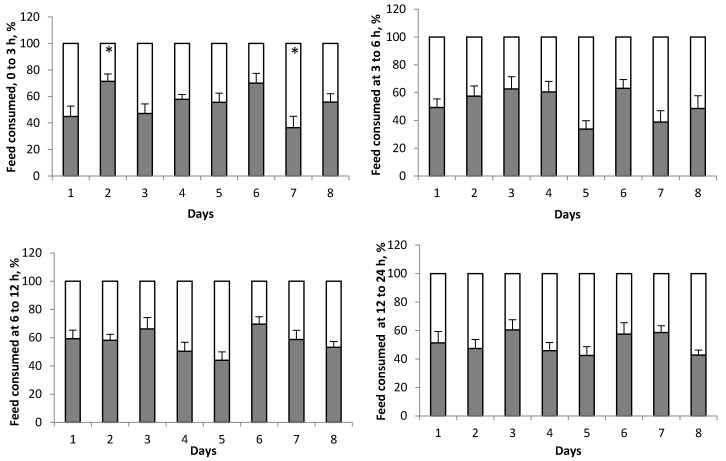
Proportions of a grain diet without (white portion of the bars) or with 3-NOP (black portion of the bars) consumed at 0 to 3, 3 to 6, 6 to 12, and 12 to 24 h after feeding during the preference period (*n* = 9; Exp. 2). Day 1 to 8 corresponds to the 8 d preference period. The symbol * indicates that proportion of CON diet consumed minus proportion of 3-NOP diet consumed within day is not equal to 0 (*p* < 0.05). No linear change in intake of 3-NOP diet, as a percentage of total daily dry matter intake, was observed over the 8 day preference period for any of the times after feeding.

In Exp. 2, average DMI over 7 days was actually greater (*p* < 0.01; Table 3) for the dNOP diet compared with the CON diet during the preference period, with greater proportion of dNOP compared with CON (*p* < 0.05) on days 2 and 6 (Figure 3). No difference in feed consumption rates between CON and dNOP was observed (Table 4) except that the consumption of the dNOP diet from 6 to 12 h after feeding was greater (*p* < 0.01) than that of the CON diet. Feed consumption at different time periods after feeding were not different between CON and dNOP during the preference period (Figure 4) except that the amount of feed consumed from 0 to 3 h after feeding was greater (*p* < 0.05) on day 2 but lower (*p* < 0.05) on day 7 for dNOP vs. CON.

## 4. Discussion

We hypothesized that a decrease in feed intake can occur due to changes in the organoleptic property of the diet when supplemented with 3-NOP. To test the hypothesis, feed preferences of beef cattle were examined by allowing animals free access to diets with or without 3-NOP. We assumed that preference of animals for CON and against dNOP was an indication of changes in the organoleptic properties of diets due to 3-NOP supplementation and could help explain short-term differences in DMI of diets supplemented with 3-NOP in previous studies. In previous studies, changes in DMI have been variable when animals were fed a diet supplemented with 3-NOP. Romero-Perez et al. [3] observed a linear decrease in DMI of beef heifers fed a high forage diet supplemented with 3-NOP at 30 to 180 mg/kg dietary DM, suggesting a possible palatability issue due to 3-NOP. Feedlot studies with beef steers conducted by Vyas et al. [1] showed a decrease or a tendency for decreased DMI for 3-NOP diets (100 and 200 mg/kg of dietary DM) during backgrounding and finishing phases, respectively. No effect of feeding 3-NOP on DMI is usually observed in dairy cows [2,12], but the dose used is considerably less (40 to 80 mg/kg DM). The lower concentration of 3-NOP in diets fed to dairy animals and difference between breed of animals (dairy vs. beef), may be, at least in part, cause for the observed variability.

No difference in DMI between the pre-preference period and the preference period was expected in the current study because both the CON and 3-NOP diets were provided for ad libitum intake. This means that even though the dNOP was preferred less than CON diet during the preference period in Exp. 1, the freely available CON diet was consumed instead of the 3-NOP diet so that daily DMI was maintained. In addition, DMI between the two feed bunks in Exp. 1 and 2 was not different during the pre-preference period indicating that animals did not have a preference for a particular bunk, thus any differences in DMI and feed consumption between CON and dNOP that occurred during the preference period were assumed to be caused by 3-NOP supplementation of the diet (i.e., CON vs. dNOP).

During the 7 day preference period of Exp. 1, average DMI was lower for dNOP compared with CON indicating that animals preferred to eat CON rather than dNOP. This decrease in DMI for dNOP vs. CON was particular evident from 0 to 12 h after feeding, the period when most of the feed was consumed. Beef cattle generally consume about 80% of feed offered (as-fed basis) within 12 h after feeding when fed once daily in confined conditions [6,8]. However, a significant interaction of diet by day for DMI during the preference period occurred because the diet preference for CON and against dNOP observed at the beginning of the period changed over the 7 days. Although preference against the 3-NOP diet existed at the beginning, the preference diminished gradually over 7 days of the preference period with no difference in DMI between treatments observed on day 5, 6, and 7 of Exp. 1. This finding indicates that animals identified the difference between CON and dNOP immediately when they did not have any prior exposure to 3-NOP. However, animals quickly acclimatized to the dNOP diet and did not show any preference between CON and dNOP within 7 days.

A gradual increase each day in the proportion of dNOP consumed 0 to 12 h after feeding occurred during the preference period in Exp. 1. To our knowledge, only one study with dairy cows has examined preference between a diet without and with 3-NOP [13]. In that study, cows received 0 and 30 mg/kg DM of 3-NOP for 6 days during which preference was determined. Then, the level of 3-NOP was gradually increased from 30 to 60, 60 to 90, and 90 to 120 mg/kg DM in 6 d intervals with preference established at each dose level. The DMI of diets with 3-NOP at 30, 60, and 90 mg/kg DM was actually greater than for diets without 3-NOP, although no difference in DMI between diets with and without 3-NOP at 120 mg/kg DM was observed (i.e., interaction of 3-NOP level by time). Therefore, the study by Melgar et al. [13] concluded that no preference between a diet without and with 3-NOP existed, which is discrepant to our results in Exp. 1. In the study by Melgar et al. [13], feed consumption was reported on a DM rather than as-fed basis, as was the case in our study, but this difference was unlikely to have caused the discrepancy because DM content was similar for diets and orts in the current study (see the footnote in Table 4). The gradual increase in 3-NOP used by Melgar et al. [13] may have helped the cows to acclimatize more easily to the 3-NOP diet, resulting in lack of preference. Also, as discussed earlier, there might be breed differences in terms of ability to detect organoleptic properties of diets and the effectiveness of methane mitigation by 3-NOP supplementation differs between dairy cows and beef cattle [14].

Differences in feed intake can also occur due to energy status [5] and this cannot be ruled out as a potential factor that resulted in the eating preference against dNOP in the current study. However, the two experimental diets (i.e., CON vs. dNOP) were almost identical except for the inclusion of 3-NOP (100 mg/kg DM) and the diets were formulated to provide sufficient energy for growing beef cattle. Our companion study with the same animals fed the same high forage diet (supplemented with or without 3-NOP at 100 mg/kg DM) that was conducted immediately after Exp. 1 measured enteric methane production and rumen fermentation [11]. In that study, the 3-NOP diet reduced methane production by 17% (181 vs. 220 g/d; *p* < 0.01). Molar proportion of propionate was increased from 20.0% to 22.7% (*p* < 0.01), which decreased the ratio of acetate to propionate from 3.3 to 2.7 (*p* < 0.01). The 17% decrease in methane production may have slightly increased the supply of metabolizable energy for dNOP vs. CON, albeit the additional net energy supply would likely have been biologically trivial [15]. In addition, an increase in propionate production has been known to lower feed intake of ruminant animals [5] and could have been a factor for the preference observed in Exp. 1. Propionate is absorbed from the rumen and metabolized in the liver and thus it is possible that the tissues needed time to adjust to a change in propionate supply when diets with 3-NOP were provided. The proportion of dNOP consumption gradually increased during the preference period in Exp. 1, although the total DMI (sum of intake of CON and dNOP) was not different from DMI during the pre-preference period. In Exp. 2, the proportion of CON and dNOP consumed was similar (i.e., no preference; see more discussion about Exp. 2 later) during the preference period, and DMI during the preference period was not different from that during the pre-preference period. If increased rumen propionate production caused the preference against dNOP in Exp. 1, DMI should have gradually decreased as the consumption of dNOP increased and DMI during the preference period should have been lower in the preference period compared with the pre-preference period in Exp. 1 and 2. This was not the case. Therefore, we accept the hypothesis that there were changes in the organoleptic properties of a forage-based diet supplemented with 3-NOP and this resulted in eating preference against dNOP in Exp. 1. However, animals rapidly acclimatized to the dNOP diet.

In Exp. 2, we concluded there was no preference against dNOP, and in fact animals consumed a greater amount of dNOP (7.6 vs. 6.1 kg/d) than CON during the preference period with a diet by day interaction (Table 3). Comparisons between proportion of CON and dNOP consumption on individual days showed significant differences with preference for dNOP on day 2 and 6 during the preference period. Although not significant, proportion of dNOP consumed was lower than 50% on day 5 and 7. Therefore, we conclude that there was no consistent eating preference for or against a diet containing 3-NOP in Exp. 1. The selection of CON and dNOP appeared to be random in Exp. 2 as the preference for dNOP was very subtle or non-existent on some days. The random selection without preference between CON and dNOP is also supported by the lack of difference between the proportions of CON and dNOP consumed at different time periods after feeding during the preference period. Large day-to-day variation in feed preference between CON and dNOP observed at each time after feeding further supports the random selection between CON and dNOP without preference. Therefore, we accept the hypothesis that animals have no or minimal preference between CON and dNOP when fed a high-grain diet if previously exposed and acclimatized to 3-NOP (i.e., Exp. 1). The results indicate that the cattle quickly acclimatized to the dNOP diet within 7 days during Exp. 1 and thereafter animals had no preference between CON and dNOP during Exp. 2.

It is important to note that the animals used in Exp. 2 had prior exposure to 3-NOP, i.e., animals were first exposed to 3-NOP during Exp. 1 and the same animals were used in another experiment that assessed 3-NOP supplementation between Exp. 1 and 2 [11]. Even though the animals were transitioned from a high forage to high grain diet without 3-NOP over 2 months, they seemed to remember the previous experience of 3-NOP as they did not need to acclimatize to 3-NOP when added to the high grain diet. It is important to state that, unlike Exp. 1, Exp. 2 did not examine preference of a diet without and with 3-NOP in animals without previous exposure to 3-NOP. The preference of cattle fed a high forage diet followed by a high grain diet supplemented with 3-NOP provides practically valuable information because standard feedlot beef production in the US is that cattle receive backgrounding diets followed by finishing diets. The target use of 3-NOP may be primarily during the backgrounding stage when more methane is produced [1] or during both backgrounding and finishing stages, where finishing cattle would have prior exposure to 3-NOP.

Our conclusion that cattle rapidly acclimatized to 3-NOP and thereafter showed no preference between CON and dNOP may be limited only to the dosage level used in the current study, i.e., 100 mg/kg dietary DM. Vyas et al. [1] reported no decrease in methane production when a high forage or high grain diet supplemented with 3-NOP at 100 mg/kg dietary DM was fed to beef cattle while NOP at 200 mg/kg dietary DM considerably decreased methane production although DMI was decreased. Therefore, the observed rapid acclimation to the 3-NOP diet (within 7 d of feeding 3-NOP in Exp. 1) and the lack of preference between CON and dNOP during Exp. 2 may have been due to the low dose of 3-NOP used. Preference studies using higher levels of 3-NOP in diets will be necessary to confirm our results.

## 5. Conclusions

Dry matter intake of beef cattle was not affected when they were offered the diets supplemented with and without 3-NOP (100 mg/kg of dietary DM) compared with only the diet without 3-NOP (i.e., pre-preference vs. preference period), regardless of type of diet (high forage and high grain) in this short-term study. However, animals that had not been exposed to 3-NOP (Exp. 1, high-forage diet) immediately recognized the difference between CON and dNOP when offered a choice, with less preference for a diet with 3-NOP. The animals rapidly acclimatized to the diet containing 3-NOP and had no preference between CON and dNOP within 7 days. Similarly, no consistent strong preference between a high grain diet with and without 3-NOP was observed during Exp. 2, possibly because the animals had previously consumed diets supplemented with 3-NOP. We conclude that the effects of 3-NOP (100 mg/kg dietary DM) on organoleptic properties of high forage and high grain diets are transient over time.

## Figures and Tables

**Table 1:** Ingredients and chemical composition of the high forage and grain diets.

Items	Basal Diets	
	High Forage Diet	High Grain Diet
Ingredients (DM, %)		
Corn silage	64.4	9.8
Distillers grain	10.2	10.0
Corn grain, ground	12.9	1.0
Whole shelled corn	-	72.8
Soybean meal	8.5	2.1
Urea	-	0.1
Limestone	1.9	1.5
Trace mineral mix ^1^	1.5	1.5
Minerals and vitamin mix ^2,3^	0.6	0.8
Chemical composition ^4^ (% of DM)		
DM, % as-fed basis	48.2	80.0
OM	92.7	94.3
CP	13.6	12.7
NDF	28.3	14.6
ADF	17.2	5.0
Ca	0.67	0.7
P	0.42	0.5

^1^ The premix contained (as-is basis) 39.3% of sodium, 53.2% of chloride, 70 mg/kg of cobalt, 400 mg/kg of copper, 70 mg/kg of iodine, 1750 mg/kg of iron, 2800 mg/kg of manganese, and 3500 mg/kg of zinc (Morton Salt Inc., Chicago, IL, USA). ^2^ Copper sulfate, 40 mg/kg; zinc sulfate, 30 mg/kg; sodium phosphate, 0.51%; vitamin A, 10 mg/kg; vitamin D, 10 mg/kg; vitamin E, 700 mg/kg in the high forage diet DM. ^3^ Calcium phosphate, 0.5%; magnesium sulfate, 30 mg/kg; Potassium chloride, 0.15%, Zinc sulfate, 20 mg/kg; copper sulfate, 10 mg/kg; vitamin A, 10 mg/kg; vitamin D, 10 mg/kg; vitamin E, 500 mg/kg in the high grain diet DM. ^4^ DM, dry matter; OM, organic matter; CP, crude protein; NDF, neutral detergent fiber; ADF, acid detergent fiber; Ca, calcium; P, phosphorus.

**Table 2:** Effects of a high forage (Exp. 1) or high grain diet (Exp. 2) supplemented with or without 3-nitrooxypropanol (3-NOP) on short-term dry matter intake in beef steers (*n* = 9).

Dry Matter Intake (kg/d)	Period ^1^		SEM	*p*-Value ^2^	
	Pre-Preference	Preference		Per	Per × day
Experiment 1	8.0	7.9	0.25	0.71	<0.01
Experiment 2	12.4	13.6	0.72	0.26	<0.01

^1^ Pre-preference, a diet without 3-NOP was delivered to two bunks in each stall; preference, a diet without 3-NOP (100 mg/kg DM) was available in one of the two bunks and a diet with 3-NOP was available in the other bunk in each stall. ^2^ Per (period), pre-preference vs. preference; per × day, interaction of period by day.

**Table 3:** Bunk and diet preference of beef steers (*n* = 9) fed a high forage (Exp. 1) or high grain diet (Exp. 2) supplemented with or without 3-NOP.

Pre-Preference ^1^	Diet		SEM	*p*-Value ^3^	
	Left Bunk	Right Bunk		Diet	Diet × day
DMI, kg (Exp. 1)	3.92	4.09	0.137	0.17	<0.01
DMI, kg (Exp. 2)	6.15	6.30	0.417	0.66	0.77
**Preference ^2^**	**CON**	**dNOP**			
DMI, kg (Exp. 1)	5.19	2.69	0.204	<0.01	<0.01
DMI, kg (Exp. 2)	6.09	7.55	0.420	<0.01	<0.01

^1^ Left and right, position of bunks in each stall; a diet without 3-NOP was available in two bunks during the pre-preference period. ^2^ CON, a diet without 3-NOP; dNOP, a diet with 3-NOP (100 mg/kg DM); the CON diet was available in one of the bunks and the 3-NOP diet was available in the other bunk in each stall during the preference period. ^3^ Diet, left vs. right for pre-preference and CON vs. dNOP for preference; Diet × day, interaction of bunk by day for pre-preference and diet by day for preference.

**Table 4:** Feed consumption rates and preference of beef steers (*n* = 9) for a high forage (Exp. 1) or grain diet (Exp. 2) supplemented with or without 3-NOP (*n* = 9).

Feed Consumption, kg as-fed	Diets ^1,2^		SEM	*p*-Value ^3^	
	CON	dNOP		Diet	Diet × day
Experiment 1					
0 to 3 h	2.69	1.27	0.150	<0.01	<0.01
3 to 6 h	2.01	0.89	0.147	<0.01	<0.01
6 to 12 h	3.93	2.05	0.223	<0.01	<0.01
12 to 24 h	2.16	2.29	0.222	0.69	0.30
Experiment 2					
0 to 3 h	2.42	2.85	0.255	0.16	<0.01
3 to 6 h	1.32	1.60	0.140	0.17	<0.01
6 to 12 h	2.51	3.42	0.247	<0.01	<0.01
12 to 24 h	1.11	1.26	0.156	0.19	0.12

^1^ CON, a diet without 3-NOP; dNOP, a diet with 3-NOP (100 mg/kg DM); the CON diet was available in one of the bunks and the 3-NOP diet was available in the other bunk in each stall (preference period). ^2^ Dry matter of TMR, 47.9% and 47.8% for CON and dNOP respectively in Exp. 1 and 81.8% and 82.2% for CON and dNOP respectively; DM of orts, 49.7% and 51.9% for CON and dNOP respectively in Exp. 1 and 81.0% and 81.3% for CON and dNOP respectively. ^3^ Diet, CON vs. dNOP; diet × day, interaction of diet by day.
